# Paramilitaries, parochialism, and peace: The moral foundations and personality traits of Slovenskí Branci

**DOI:** 10.1371/journal.pone.0281503

**Published:** 2023-03-21

**Authors:** Pavol Kosnáč, Justin E. Lane, Monica Duffy Toft, F. LeRon Shults

**Affiliations:** 1 DEKK Institute, Bratislava, Slovakia; 2 Department of Political Science, Masaryk University, Brno, Czech Republic; 3 CulturePulse, Inc., Middletown, Delaware, United States of America; 4 Slovak, Bratislava, Slovak Academy of Sciences, Bratislava, Slovakia; 5 The Fletcher School, Tufts University, Boston, Massachusetts, United States of America; 6 Institute for Global Development and Social Planning, University of Agder, Kristiansand, Norway; University of Massachusetts Lowell, UNITED STATES

## Abstract

Paramilitary organizations have increasingly become a cause for concern among policy makers and the media in recent years, in part because the former are often seen as a potential threat to peace (or at least to the status quo of the current political systems) in the countries in which they emerge. Organizations such as the Oathkeepers and 3 Percenters (also known as III%ers) in the United States have grown significantly in the last two decades, while paramilitary organizations playing a key role in both offensive and defensive actions in Crimea and the Donbas Region have become a focus of discussion in the Russian war on Ukraine. Although they have not always garnered as much attention, paramilitary organizations in Central and Eastern Europe have a long history. While most are relatively inactive, others play a wide variety of active roles, sometimes even running operations in parallel with a state’s official armed forces (e.g., the PMO serving the state in Poland, or the Night Wolves helping Russia capture Crimea). Despite the increase in the number and activity of these paramilitary organizations, little is known about the personal, social, moral, and psychological background of the individuals who join them. After reviewing the history and ideology of the largest paramilitary organization in the Slovak Republic, this article presents and discusses the results of a survey administered to the group. This survey used different measures of personality, morality, and identity, as well as information about respondents’ personal background, family history, socio-economic status, and political ideology. We find significant relationships between certain individual personality traits and the importance of certain moral foundations among members of these organizations in relation to their broader social community.

## Introduction

In recent years, the activities of paramilitary organizations have become increasingly high profile and many national governments have now devoted significant resources to tracking their growth and activity. Despite this increased attention, there is little research that seeks to identify and understand what motivates individuals to join such groups and operate within them, especially in European and other developed countries. Populations in the latter typically experience comparatively high qualities of life within relatively stable social, economic, and political environments. Joining a paramilitary organization requires sacrificing personal time and resources in order to help the group achieve its objectives. Participation often includes basic training routines of armed forces such as light infantry drills, scouting skills in urban and natural environments, annual 7-day boot camps where adult men, and occasionally women, allow others to shout at them, all of which causes high levels of physical and psychological stress and sleep deprivation. Joining such groups can also involve participating in rituals of shaming and bonding, during which one’s personal identity is deconstructed and then reconstructed to fit more closely with ideals, beliefs, and values of the paramilitary organization. Moreover, belonging to the latter brings little prestige in wider society because members are often viewed as dangerous outcasts, fascists, or right-wing extremists. Why, then, would individuals join and maintain membership in such a group?

Here we present the findings of a study of one of the largest active paramilitary organizations in Central and Eastern Europe, a group based in Slovakia called *Slovenskí Branci* (trans. “Slovak Conscripts”). As a result of our unprecedented access to this group, we have been able to carry out research that uses key psychological measures to help us to understand the socio-cognitive profile of individuals willing to make personal sacrifices to join the paramilitary organization. As explained in more detail below, our approach combined original field research with a psychometric survey that measured members’ moral foundations, individual and group identities, personality profiles, as well as aspects of their social and political beliefs.

The data reported here contribute to a deeper understanding of the relationship between paramilitary organizations and social instability and potential threats to peace at the national level. This is an issue of growing concern in several societies, especially in light of the steady decline of social cohesion in many European countries, including Slovakia [1], plummeting trust in institutions [2], low political participation [3], and the emergence of alternative ideologies and socio-cultural echo-chambers that contribute to polarization in political discourse [4–6].

### Slovenskí Branci and paramilitary ideology in Central and Eastern Europe

There is no universally accepted conceptual framework of paramilitarism or consensual definition of a paramilitary group. In Central and Eastern European (CEE) the term “paramilitary” is primarily limited to academic discourse, while the term “domobrana” (home guard), which has a similar meaning as the English “militia,” is more popular in common parlance. The Oxford English Dictionary offers this general definition: “A paramilitary organization is a semi-militarized force whose organizational structure, tactics, training, subculture, and (often) function are similar to those of a professional military, but is not formally part of a country’s armed forces.”

Paramilitary organizations are not new to Slovakia. In fact, Slovakia has seen different kinds of paramilitary groups emerge during the last century. These included groups as diverse as the unarmed Czechoslovak sport-patriotic clubs (e.g., “Sokol” and “Orol”) that sprung into existence in the last 50 years of the Austro-Hungarian Empire, the fascist Hlinka´s Guards, and later the Peoples Militias, the political paramilitaries of the Czechoslovak Communist Party. Nevertheless, in the last 30 years Slovakia had almost no state-organized paramilitary activity. Most paramilitary groups that originated during this period were small groups organized by far-right sympathizers that typically lasted no longer than a few years and counted only about a dozen core members. These militias were closer to urban combat type units than paramilitary organizations possessing general military skillsets [7]. Slovenskí Branci is the first serious, relatively large, and enduring paramilitary organization in Slovakia to emerge in the last three decades.

Slovenskí Branci was founded in Slovakia in 2012 by four individuals, only two of whom (Peter Švrček and Michal Feling) remain in the group today. When they founded the group, they were 16 years old and it was sort of a lark or a hobby to the young men. The decision to establish the organization was somewhat spontaneous, inspired by stories of their grandfathers’ time in (compulsory) military service and their own engagement in the Airsoft community (a game similar to paintball). One of the founders also served in the Slovak armed forces and had romanticized ideas concerning military environments. Over the past decade the organization has grown and thrived, attracting serious attention from both financial sponsors and the national security structures of Slovakia and neighboring countries. This concern was in part driven by their success in inspiring others to join the group and their early contact with representatives of the Slovak Revival Movement (SHO, “Slovenské hnutie obrody” in Slovak), a far-right political organization with political ambitions.

The SHO appeared to influence early members of Slovenskí Branci. Both groups accepted the SHO’s interpretation of the history of WWII, which holds that, under the president of the first Slovak state (Jozef Tiso), the government made deals with the Third Reich that “protected” Slovakia from communism (communism is despised by the SHO). This is in stark contrast to the Russian narrative that the Communists, under Stalin, liberated Slovakia from Nazi occupation. The inspiration for the ideology of the SHO also extends further back in time, revolving around a romanticized vision of the Slovak nation that idealizes the role of 19^th^ century Slovak national heroes who shed their blood for the future of their fellow Slovaks.

Despite the SHO’s anti-Russian attitudes regarding the events of WWII, they have had a good relationship with several Russian organizations, including the “Association Stjag,” which united several Cossack-led paramilitary organizations that organized summer camps for the Cossack youth. The latter were trained in traditional Cossack martial arts that included typical military skills of light infantry and reconnaissance, as well as cultural skills such as horseback riding, physical hardening practices such as ice bathing and fasting, innovative punishments, “Slavic combat games,” and the use of bladed weapons and whips.

The SHO helped the original members of Slovenskí Branci to secure several places in a training camp near Moscow in summer of 2012, where they underwent military-style training over the course of several weeks. This experience was transformative in contributing to a sense of mission for Slovenskí Branci and in developing and deepening a culture around militaristic activity and political activism. This training came at a crucial time, after the influence of the Slovak Revival Movement had largely disappeared, which left Slovenskí Branci as the most visible organization promoting a vision of what it means to be a true “Slovak” besides the Slovak National Party (SNS). At the same time, Slovenskí Branci also broke from the previous SHO interpretation of Tiso’s government and instead of honoring his decisions (visibly shown through standing guard over his grave), they shifted toward honoring Slovak anti-fascist fighters such as the Slovak Partisani (guerrilla resistance fighters against the Nazis) and Milan Rastislav Stefanik—a WWI hero and one of the founding fathers of the Czechoslovak state. Today, the leaders of Slovenski Branci think of their earlier stance towards the Nazis and Jozef Tiso’s government as “far-right leaning” and “mistakes of [their] youth.” Nevertheless, as we discuss later, the organization is still considered “right-wing,” as evidenced, for example, in their voting patterns.

Slovenskí Branci adheres to a strict military ethos, utilizing a hierarchical structure with leaders and founders at the top of the organization. Peter Švrček is the President and *de facto* leader, while Michal Feling is second in command and the “field” commander in charge of military doctrine. They preside over the “Council of Slovenskí Branci” consisting of commanders of local units, representing towns and county seats. Council members hold the title of “starešina,” meaning “old man” in an archaic Slovak dialect; this is an advisory position typically granted to older members. The number of Council members fluctuates between 6–8 individuals. There are currently three regiments—East, West and Middle. The two largest, most active, and most professional units of the organization have approximately 30 members each and exist in the mid-sized cities of Trnava and Nitra—in the West of the country, close to the Austrian Border.

Active membership of Slovenskí Branci has fluctuated between 100 and 200 members in the last five years. Currently the core group has roughly 130 formal members, of which about half partake in regular training activities. The COVID-19 pandemic has slowed down the activities of the group, though according to leadership the events surrounding the pandemic have driven public interest in the group and enhanced recruitment.

The organization has claimed to run up to 17 units in different localities around Slovakia, including groups abroad in Moravia, Czech Republic, that were never formally considered members. They have had training groups throughout the country and have recently established training barracks in the area of Košice (in the east of Slovakia).

The most common activity of Slovenskí Branci is providing ongoing paramilitary training to Slovak citizens. This typically happens on a bi-weekly basis. Over the last decade, the leadership has also devised a year-long basic training program, which culminates with a rigorous test during a week-long bootcamp each summer. Every commander is issued a manual that contains the training regimen, and the leadership of the group maintains careful supervision of all activities.

Members of Slovenskí Branci consider themselves part of something that is more than merely a paramilitary unit; they are a nationalist organization with a mission to revive patriotic traditions of Slovakia. This is reflected in the way in which the group organizes activities outside the traditional military domain such as repairing historical cultural sites (the leader and co-founder is an archeologist by training), planning remembrance events at graves and monuments of Slovak historical figures, cleaning illegal dump sites, and serving as organizers and volunteering as staff during sporting and cultural events (e.g., folklore festivals at local castles). They also regularly join relief teams during the flood season or in winter during weather emergencies and participate in search and rescue operations organized by local authorities.

However, some of the group’s activities are considered controversial and have attracted the attention of the national media and state security apparatus; such activities include giving lectures about Slovak history and organizing “defense education” at elementary schools, sending “monitoring squads” to Roma villages in Eastern Slovakia, and patrolling the borders of refugee camps in Slovakia and refugee routes at the Slovak-Austrian and Slovak-Hungarian borders during the refugee crisis of 2015–2016. Perhaps the most controversial aspect of Slovenskí Branci is its apparent link to Russian operatives, especially the Night Wolves, a Russian paramilitary motorbike gang that participated in the 2014 conflict in Crimea [8] and Donbas [9]. Many public actions of Slovenski Branci are related to various anti-system protests. However, when appearing in public wearing Slovenski Branci colors, members typically portray themselves as “peacekeepers” and present themselves as a security alternative, rather than as “stirring up” conflict.

The ideology and mission of the group has recently been clarified in a statement by one of their founders, Peter Švrček:

The goal of Slovenskí Branci is to minimize the impact of any crisis situation on the civilian population. … Why are we doing this? First of all, the Slovak Armed Forces are in ruins, they are no longer even formally fulfilling their obligations—it was exempted from the soldier’s oath that his job is to defend the civilian population. At the same time, we can observe the decline of the value compass of today’s youth, so we have combined several needs here. We do not have a compulsory military service anymore, young people lack it, and at the same time one of the basic pillars of statehood and existence of every nation is the ability to defend itself. We know that no organism will survive without a defense mechanism. We have offered to help create such a mechanism, within our modest means, when our armed forces are in the state they are in, and they also reflect the state of society as such… [10].

Members of Slovenskí Branci insist that they do not adhere to any one political ideology. They claim they are members of an apolitical, patriotic self-defense organization that emulates the historical and cultural tradition of Slovak volunteer forces that fought for freedom (e.g., the Czechoslovak legions, the sport-defense civil organizations of the First Czechoslovak Republic “Sokol”, and the anti-fascist partisan fighters of WWII). The political rhetoric of the group resembles U.S. militia organizations that state that they do not organize against any concrete enemy (e.g., https://thethreepercenters.org/about-us/). Slovenskí Branci exists to create a self-support network that can be utilized in any type of crisis that might emerge on Slovak territory. Based on their historical experience that in order for a nation to remain free, it should have an organized and trained self-defense force, they argue that the government should support and not hinder or criminalize such activities As with other nationalist organizations, self-defense of the homeland and territoriality are critical components of their ideology [11]. Within Slovenski Branci, there has not been an official “enemy” identified. In other words, there is no single outgroup that appears to be identified as the perpetuator of threats perceived within the group. The leadership focuses primarily on the negative consequences of the crumbling of civil order, implying that the de facto “enemy” is anyone who contributes to that crumbling. This animosity can be focused on groups such as migrants, but it can also just as easily become focused on native Slovaks who might attempt to take advantage of civil unrest.

Despite Slovenskí Branci being officially apolitical, members do not hide their preference for a certain set of political values. Early in their history, they may have had “far-right” values, but these shifted to a pan-Slavic and strongly anti-establishment value set. More recently, the values of nationalism and patriotism have emerged as more prominent. However, how “nationalism” is defined and what is viewed as best for the nation has evolved over time along with changes in the ideology and interpretation of history by the group leadership. Other strong sentiments held by a majority of members include suspicion towards what is considered “new” and “foreign” influences brought in by the media and cosmopolitan urban elites, distrust of international institutions, especially the European Union and North Atlantic Treaty Organization (NATO), and disgust over the “sunshine mentality”—a Slovak equivalent of the derogatory moniker “snowflake” in the US—indicating an overly sensitive and politically correct person, typically aligned with strong liberal and inclusive political views. While many of these values and beliefs appear to align well with the current Russian narrative (e.g., pan-Slavic identities, distrust of NATO), it is important to note that when Russia first invaded Ukraine, the leadership of Slovenski Branci denounced the invasion as an act of aggression. This suggests that the values of paramilitary groups should be understood as complex and multifaceted, rather than as based on a simplistically polarized political framework. Although the group’s values do have some overlap with that of Russia, ultimately its core value of protecting Slovak sovereignty and security played a more central role in the group’s alignments since the Russian invasion of Ukraine in 2022. This is likely because Russia’s actions are viewed (in some way) as an attempt to repeat a version of the 1968 invasion of Czechoslovakia.

Currently, Slovenski Branci are not considered illegal. They have never formed any legal body, and any legal operations are connected to the leaders as individuals, or different friendly NGOs. The common perception of Slovenski Branci among the public appears to be that they are far-right wing militants. They are often associated by the general public with fascist or neo-nazi parties, despite having a variety of backgrounds (including members from ethnic/racial minority groups). It would be safe to say that, due to coverage by several national news sources, the group is well known throughout the region by the general public. Government organizations are aware of, and monitoring, the group’s activities.

### Theoretical framework

While sociological, political, and economic analyses of the groups such as Slovenskí Branci are crucial for understanding the contextual factors at work in their emergence and expansion, our analysis here is driven primarily by insights from empirical findings and theoretical developments in personality and social psychology. We believe that understanding the character traits and driving motivations of individual members can complement other sorts of analyses, providing unique policy-relevant insights into the potential actions of the group as a whole.

As mentioned above, there are no ready-made theories of paramilitary psychology from which we can draw. However, there are cross-culturally applicable psychological frameworks and validated empirical measures for morality, nationalism, values, and identity, which can be taken as theoretical guides for understanding Slovenskí Branci and its potential role in future conflicts or peace building. In our attempt to better frame the ideology and culture of Slovenskí Branci, we are drawing mainly on three theories: Moral Foundations Theory, the Big-Five Personality Theory, and Identity Fusion Theory.

#### Moral foundations theory

Moral Foundations Theory (MFT) was devised to explain variation in human moral judgements and reasoning [12], and has been used to study cultural and political differences between different groups. MFT suggests that there are at least five largely separate, possibly modular, moral domains with which individuals regularly engage [13]: Harm, Fairness, Ingroup (Loyalty), Authority, and Purity. These foundations have been summarized as follows:

Individualizing domains:

1. Harm: “The suffering of others, including virtues of caring and compassion.”
2. Fairness: “Unfair treatment, cheating, and more abstract notions of justice and rights.”

Binding domains:

3. Ingroup loyalty (also referred to as “loyalty” in the literature, but here we refer to it as just “Ingroup” so as to not confuse with the domain of authority): “Obligations of group membership” such as “self-sacrifice, and vigilance against betrayal.”
4. Authority: “Social order and the obligations of hierarchical relationships, such as obedience, respect, and the fulfillment of role-based duties.”
5. Purity: “Physical and spiritual contagion, including virtues of chastity, wholesomeness, and control of desires” [14].

Earlier research [15] has found that liberals and conservatives largely focus on different moral values. Later survey research has both replicated this finding, and found exceptions to it [16]. Overall, the research in MFT suggests that liberals tend to focus on the “individuating” moral domains of fairness and harm (which focus on individual social interactions), while conservatives also emphasize the “binding” moral domains of loyalty, authority, and purity (which help to define ingroup-outgroup boundaries). Often, traditional traits that we associate with different aspects of the political spectrum, are related to these domains. For example, religiosity, which is often related to conservatism in wider cultural dialogues, is related to purity in that purity concerns appear to drive religiosity, but religiosity does not appear to drive connections with purity in larger social movements [17]. However, in Slovakia, as in many cultural contexts in Eastern Europe, the line between religion and ethnicity or nationality are blurred. Catholicism has been the dominant religious tradition in the country for centuries, and it is often assumed that if one is Slovak that they are also Catholic, and many cultural traditions and celebrations are deeply rooted in religious belief schemas. As such, it is difficult to disentangle religion from ethnicity in Slovak culture, but it is relevant to note that there is a tacit assumption that Slovenski Branci, being a more right-wing organization, are more religious in their moral reasoning.

These findings from the MFT literature could lead us to expect that members of Slovenskí Branci will have generally high ratings across all five moral domains (consistent with a conservative moral signature). However, given the strength of their group ties, we might wonder whether they would score more highly in the “binding” compared to the “individualizing” domains. In other words, we might expect members to be more willing to advance group interests over their individual interests. This informs our first hypothesis:

H1—*There will be no significant differences between the moral domains of authority*, *ingroup and purity*, *and none of these three “binding” domains will be significantly lower than the “individualizing” domains of harm and fairness*.

#### Big-five personality theory

As with MFT, the Big-Five Personality Theory (BFPT or five-factor model) is a taxonomy of traits (Goldberg, 1992). These traits are:

1. Openness: focusing on imagination, curiosity, and cautiousness
2. Conscientiousness: focusing on efficiency, organization, and carelessness
3. Extraversion: focusing on sociality, energy, and reservation
4. Agreeableness: focusing on friendliness, compassion, and criticality, and
5. Neuroticism: focusing on sensitivity, nervousness, and resiliency

Previous research has found that individuals who endorse extremist groups tend to be less agreeable, less neurotic, and more open than non-extremists [18]. We expect to find similar traits among members of *Slovenskí Branci*. This informs our second hypothesis:

H2—*Slovenskí Branci members will have relatively low levels of agreeableness and neuroticism and relatively high levels of openness*.

In addition, we suggest an exploratory hypothesis (H2b) that combines aspects of H1 and H2. Specifically, we investigate the extent to which personality factors are related to the perceived importance of different moral domains within the group.

H2b –*The predicted high levels of agreeableness and neuroticism (the foci of H2) will affect the binding moral values of ingroup*, *purity*, *and authority (the foci of H1)*, *while member’s levels of openness will affect their individuating values of harm and fairness*.

I

#### Identity fusion theory

To explain the potential extreme behaviors that militia members will enact or endorse, Identity Fusion Theory (IFT) is also a useful framework. IFT is a novel approach used to understand critical differences and deficiencies in the social identity literature to explain the psychological tendencies of individuals who become part of groups that are willing to go to extremes (including self-sacrifice) in defense of their identity [19–21]. This behavior occurs even when an individual’s social identity is inconsistent with creating a generally positive self-image and under intense forms of self-criticism. Although the literature suggests that some individuals will fail to identify and fuse with the group, a subset of individuals will defend his or her shared identity—sometimes to the point of fighting and dying for the group—indicating that they have fused their individual identity with that of the group [22–24].

We use IFT to frame our understanding of extremism, terrorism, and self-sacrifice in several contexts. Its key hypothesis is that highly “fused” individuals experience a “oneness” with their group such that the boundary between their personal and social identities becomes blurred. Research suggests that two key factors in the formation of identity fusion are shared negative (social) experiences and the internalization of key beliefs of one’s social schema as part of one’s personal self-schema. As such, it suggests that one factor contributing to levels of fusion among members of Slovenskí Branci is the extent to which they are socially ridiculed as racists or fascists (falsely in their mind) and the extent to which they believe that Slovakia should be self-sufficient, which is a key belief of what they belief forms a healthy Slovak identity around which they as a group have fused. This suggests a third hypothesis for the study:

*H3—Identity fusion among Slovenskí Branci will correlate with key beliefs about the self-sufficiency of Slovakia and the social group’s positive self-image*, *when controlling for other cultural beliefs*.

### Methods

This study is part of ongoing field research on Slovenskí Branci that began in 2015 and has previously utilized qualitative interviews and longitudinal participant observation of their training and teambuilding activities. The current study reports results from a survey of the organization. Access to the group was provided without any concessions except that the names of the regular members were not to be made public. We were also given access to the internal chat rooms of the group, which included the entire chat history (prior to the time of the current study). This provided us with a longer period of time to study, enabling us to evaluate possible shifts in values and interpretations of history and ideology. Receiving this level of access was initially surprising. However, we came to understand that, from the perspective of the leadership, Slovenskí Branci is not doing anything wrong; on the contrary, they believe they are advancing their nation’s interests and providing us access provided them with a way to demonstrate that they have nothing to hide.

In 2020 the group leadership agreed to allow a large survey to be distributed among its members, with more than 50% of active membership participating, including all members in leadership positions. The questionnaire consisted of three standardized tests—Big 5 Personality, using the mini-IPIP psychological questionnaire [25], the Moral Foundations Questionnaire [26], and 50 extra questions including basic demographic questions, political affiliation, and opinions on social, economic and lifestyle issues. Members of the group also filled out the full visual identify fusion scale [21, 27]. See online supplemental materials for details of all included surveys: https://github.com/DEKKinstitute/paramilitarysurvey1.

We talked to more than 100 members over the summer of 2021, but only 46 members were willing to participate in the survey (or psychometric) part of the study. It would have been optimal to have all members participate in all parts of the survey but achieving a 40% participation rate of the entire active membership is considered a representative number. Moreover, since members of all units participated, including the leadership, there are no whole clusters, membership sub-groups, or important influential members missing from the sample.

The current report focuses on presenting the findings of the moral foundations data, contextualized by the data gathered during the field research and history of the group, reported above. All instruments were deployed in Slovak.

### Ethics statement

The supervisor at the Department of Political Science of Masaryk University approved the study. Specialized ethics committees are not required for standard questionnaire research that does not collect personal or medical data. All of the participants gave written informed consent before data collection.

## Results

After data collection, results were analyzed in R [28]. Of the individuals who took part in both the qualitative and survey part of the study, the 16 participants who did not successfully pass the standard attention check of the Moral Foundations Questionnaire (MFQ) were excluded from the sample. The resulting sample (*n* = 46) was entirely male (although the group does admit female members, it currently has only male members). Key background characteristics of the surveyed group is outlined in Table 1. In the final row, it is important to note that only Bratislava (the capital) and Kosice in the East of Slovakia have populations over 100,000.

**Table 1:** Background of respondents.

Background characteristics							
Age	Under 18		18–25	25–35	35–45	45–55	>55
	1		20	14	6	5	0
Education	Basic		Secondary school, not GSCE exams	High school	University graduates		
	1		6	26	13		
Income	No income	Unemployed	Less than average	Average	Higher than average		
	9	1	4	10	22		
Marital status	Single	Married	Divorced w/children	Divorced w/o children			
	33	9	1	1			
Residence, number of inhabitants	<1000	1000–5000	5000–20,000	20,000–100,000	>100,000		
	2	15	8	13	8		

**Fig 1:**
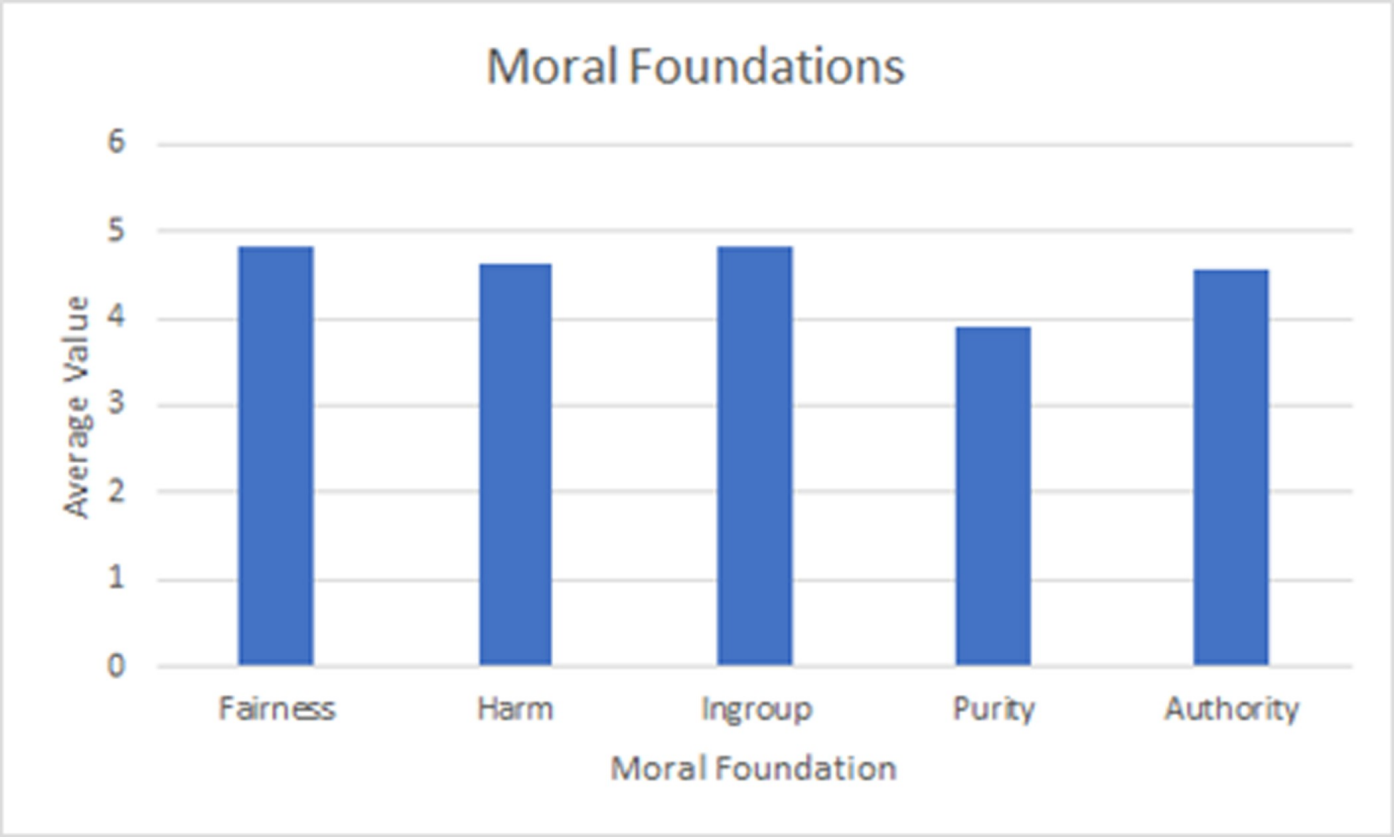
Moral foundations of the paramilitary organization.

To test H1, we utilized data collected from the moral foundations questionnaire. The respondents all completed the moral foundations questionnaire. The results revealed what could be considered a “conservative” or “right-wing” moral signature, with all the moral domains being high relative to the scale midpoint. As noted above, past research has been shown that liberal or “left-wing” moral signatures emphasize fairness and harm more than the ingroup, purity, or authority domains, while conservative signatures indicate an activation of all five domains about equally. The scale measures go from 0–6, with a midpoint of 3. The respondents displayed average values close to 5 for all moral domains except purity, which was close to 4. This suggests a generally conservative signature but with a relatively lower score on purity (Fig 1):

The means and standard deviations are presented in Table 2 below:

**Table 2:** Means and standard deviations from MFQ results.

	Average (Mean)	SD
**Fairness**	4,84	0,75
**Harm**	4,63	0,79
**Ingroup**	4,84	0,70
**Purity**	3,89	0,98
**Authority**	4,57	0,71

T-tests between each dimension revealed that purity was an outlier, with all of the other moral domain measures being significantly higher along each dimension: authority was significantly higher than purity (t = 3.81_(81.56)_, p < .01, 95%CI = [.32:1.03]), harm was significantly higher than purity (t = 3.95_(85.76)_, p < .01, 95%CI = [.36:1.10]), fairness was significantly higher than purity (t = 5.19_(83.83)_, p < .01, 95%CI = [.58:1.31]), and ingroup was significantly higher than purity (t = 5.33_(81.06)_, p < .01, 95%CI = [.59:1.30]). These findings strongly suggest that members of Slovenskí Branci have a clearly “conservative” moral signature. However, this signature is unique because it has significantly lower levels of purity than are predicted by MFT. This appears to be due to the non-religious nature of the Slovenskí Branci identity and the general emphasis of religious notions of purity within the MFT. Overall, the data supports H1.

What about H2? Results regarding the responses to the personality scale revealed that, generally, respondents were high in agreeableness, but low in neuroticism. Means and standard deviations are included in Table 3 below for each dimension.

**Table 3:** Means and standard deviations for the B-5 personality dimensions survey.

	Average (Mean)	SD
**Openness**	3.94	0.78
**Conscientiousness**	3.73	0.67
**Extraversion**	3.56	0.91
**Agreeableness**	4.15	0.75
**Neuroticism**	2.61	0.99

T-tests between each dimension revealed that there are significant differences between neuroticism and all other measures: openness (*t* = -7.17_(df = 85.16)_; *p* < .01; *95%CI* = [-1.70:-0.96]), conscientiousness (*t* = -6.40_(df = 78.64)_; *p* < .01; *95%CI* = [-1.48:-0.78]), extraversion (*t* = -4.81_(df = 89.30)_; *p* < .01; *95%CI* = [-1.35:-0.56]), agreeableness (*t* = -8.40_(df = 83.75)_; *p* < .01; *95%CI* = [-1.91:-1.18]) as well as differences between agreeableness and conscientiousness (*t* = 2.79_(df = 88.74)_; *p* = .01; *95%CI* = [0.12:0.71]) and between agreeableness and extraversion (*t* = 3.38_(df = 86.89)_; *p* < .01; *95%CI* = [0.24:0.93]). Lastly, there were significant differences between openness and extraversion (*t* = 2.15_(df = 87.93)_; *p* = .03; *95%CI* = [0.03:0.73]).

This pattern suggests mixed support for the hypothesis that Slovenskí Branci members will have low levels of agreeableness and neuroticism and relatively high levels of openness. While neuroticism was low and openness was relatively high (in support of the hypothesis), agreeableness was the highest of all measured personality factors (not in support of the hypothesis).

In order to test H2b, which was an exploratory hypothesis, five linear regressions were designed to measure the possible effects of each of the personality variables on each of the five moral foundations. It was found that only neuroticism had a significant effect on ingroup, and authority values, while only agreeableness had a significant effect on purity and harm; there were no significant effects on Fairness as a value. Results are presented in the table below (Table 4) where each of the moral foundations serve as the dependent variable for each of 5 regression models, and the personality traits are independent variables in each model. This approach serves a similar purpose as a correlation plot would, but allows for the presentation of mutual effects of personality variables on the moral domains.

**Table 4:** Regression results of the big-5 personality (as independent variable) and moral foundations (as dependent variable) surveys.

	Ingroup		Purity		Authority		Fairness		Harm		
	*β*	*p*	*β*	*P*	*β*	*p*	*β*	*p*		*β*	*p*
**Openness**	0.02	>.05	-0.18	>.05	-0.23	>.05	0.03	>.05		-0.03	>.05
**Conscientiousness**	0.21	>.05	0.14	>.05	0.08	>.05	0.31	>.05		-0.02	>.05
**Extraversion**	0.09	>.05	-0.16	>.05	-0.03	>.05	-0.22	>.05		-0.15	>.05
**Agreeableness**	0.30	>.05	0.38	.02	0.11	>.05	0.28	>.05		0.60	< .01
**Neuroticism**	0.38	.02	0.25	>.05	0.40	.02	0.20	>.05		0.12	>.05
***R2***	.15		.14		.11		.17		.25		
***F***	2.53		2.45		2.065		2.87		3.93		
***df***	5, 40		5, 40		5, 40		5, 40		5, 40		

In testing H3, we utilized several questions about socio-cultural and political beliefs of the organization’s members that can be grouped into questions about trust in institutions and economics, beliefs about gender norms, conspiracy theories, and socio-cultural aspects of Slovakia. A copy of the complete survey is included in the online supplemental materials (https://github.com/DEKKinstitute/paramilitarysurvey1). For this specific analysis, we focused on participants’ answers about socio-cultural aspects of Slovakia. Responses to these questions were given on a scale of 1–6. The questions attempted to capture the extent to which participants:

- agree that there is no objective reality
- agree that world affairs are more influenced by secret cabals rather than politicians
- are offended when people say that nationalism is racism/fascism
- believe that Slovakia should be self-sufficient
- agree that profit can be moral
- agree that individuality is better
- agree that society should dictate individual norms
- believe that meritocracies are better

The results are presented in Table 5.

**Table 5:** Testing for identity fusion with key beliefs about self-sufficiency of Slovakia (controlling for other cultural beliefs).

Variable	*β*	*SE*	*T*	*P*
Intercept	-.38	2.00	-.19	.85
Agree with postmodern viewpoints	-.07	.10	-.67	.51
Offended when nationalism is compared to racism or fascism	.38	.18	2.08	.04
Agree that Slovakia should be self -sufficient	.68	.32	2.15	.04
Agree that profit can be moral	-.17	.16	-1.08	.29
Agree that individuality is better	-.07	.10	-.71	.48
Agree that society should dictate individual norms	.04	.13	.32	.75
Agree that Slovakia should be a meritocracy	-.04	.16	-.24	.81

We utilized a linear regression to test the effect that stronger endorsement of these beliefs has on their reported levels of identity fusion among the participants, hypothesizing that key ideological statements related to self-sufficiency and being offended by the association of nationalism with fascism/nationalism will have the strongest effects on identity fusion. In our analysis we found that our two target variables were both significant (f = 2.35_(7,38)_, r^2^ = .17, p = .04).

## Discussion

The results reported here provide a rare insight into the moral foundations and psychological profiles of the members of this paramilitary organization. While we found mixed support for our specific hypotheses, all of our results (whether or not they were in line with the hypotheses) provided valuable insight into a growing socio-political phenomenon that, due to security and access concerns, is almost never studied with this level of access and detail. See Table 6 for a summary.

**Table 6:** Chart of hypotheses and results.

	Hypothesis	Result
H1	*There will be no significant differences between the three binding moral domains of authority*, *ingroup and purity*, *and none of these binding sentiments will be significantly lower than harm and fairness (the individualizing moral domains)*.	Purity was found to be significantly lower than the other moral domains. Otherwise the data suggested a standard conservative moral signature where individualizing moral domains are of relatively equal importance to the binding moral domains.
H2	*Slovenski Branci will have low levels of agreeableness and neuroticism and relatively high levels of openness*.	Neuroticism was significantly lower than all other traits. Openness was significantly higher than extraversion. Agreeableness was the highest of all measured traits.
H2b	*We expect that the predicted high levels of agreeableness and neuroticism (the foci of H2) will affect the binding moral values of ingroup*, *purity*, *and authority (the foci of H1)*, *while member’s levels of openness will affect their individualizing values of harm and fairness*.	Generally, personality was not linked to morality. Harm and purity were only affected by agreeableness, while ingroup and authority were only affected by neuroticism
H3	*Identity fusion among Slovenskí Branci will correlate with key beliefs about the self-sufficiency of Slovakia and the social group’s positive self-image*, *when controlling for other cultural beliefs*.	Beliefs related to self-sufficiency and being offended by the association of nationalism with fascism/nationalism were positively related to identity fusion.

In testing H1, we generally found evidence for our hypothesis, with the interesting exception that purity was significantly lower than the other moral values. One of the reasons for this might be that the moral foundations classification of purity is heavily directed towards ideas of religious purity, which can even be seen in the questions themselves, as question 16 of the Moral Foundations Questionnaire is “Whether or not someone acted in a way that God would approve of”. However, the field research suggests that religiosity is not a key concern among members of the group. As such, other measures of purity developed in the future that look at aspects of “social” or secular ideas of purity might be more of a general measure applicable to non-religious forms of moral purity (such as ethnic or national purity).

In testing H2, we found that neuroticism was significantly lower than all other personality traits and that openness was significantly higher than extraversion. This can be compared to the findings from Alizadeh et al. [18], which indicated that neuroticism was lower and openness was higher in extremist groups. However, while Alizadeh et al. found that extremists are typically less agreeable, the paramilitary profile uncovered here found that agreeableness was the highest scoring personality trait, followed by openness. Future research should aim to compare the results from the paramilitary sample to a comparable general population or members of non-paramilitary organizations.

In any case, the results reported here indicate that (at least these) paramilitary members do not share the same psychological profile as terrorists or other extremists, which suggests that treating paramilitary groups in the same way as terrorist groups could be a mistake for policy makers and security experts. For example, the high level of agreeableness in the paramilitary organization supports the notion that they are likely to be more open to negotiation and compromise than extremists. This suggests that negotiation tactics developed for extremists may be inapplicable for paramilitary organizations and that new policies, tactics, and negotiation scripts should be developed for dealing with these groups. In addition, should future studies find similarities between traits and beliefs of the general population and those of paramilitaries, this would shed light on public acceptance or tolerance of some groups and help stakeholders to better calculate the risks that there might be a serious conflict or security issue involving these groups as perpetrators.

In addition, future research may seek to employ new methods, specifically computer modeling and simulation, in order to better estimate the causal factors underlying the changes in membership in the group over time. The fieldwork revealed some notable reasons for individuals leaving the group. These included the group leaders’ public unveiling of a particular political ambition by appearing with the leadership figures of several political parties and alternative media personalities in Slovakia (Štefan Harabin, Vladimír Mečiar, Tibor Eliot Rostas). Some individuals left when police intervened with an annual training event, and others left when the group first began to get negative press. Overall, there appear to be two main motivations for leaving the group. The first has to do with loss aversion: leaving the group to mitigate potential personal losses by avoiding arrest or public scrutiny of their reputations. The second has to do with the social identity approach to leadership: leaving the group because its leaders do not appear to be acting in congruence with the ideology of the group. These could be the topic of future research.

When testing H2b, our results suggested that moral domains are largely unaffected by personality differences. Fairness, a moral domain shared across the political spectrum [15] was unaffected by personality traits. Harm and purity were only affected by agreeableness, while ingroup and authority were only affected by neuroticism; in all cases, these effects were positive. This is interesting as neuroticism was significantly lower than all other personality traits, while agreeableness was significantly higher than neuroticism, conscientiousness and extraversion. In addition, while previous research has found that purity is a value unique to conservatives [15, 29], our data found that purity is significantly lower than the other values, suggesting that further research is required to establish if this pattern holds in comparison with a broader conservative population, or if the patterns of moral foundations across the political spectrum demonstrated in other societies do not hold for the Slovak population. It is possible that low purity, high authority, and high ingroup concerns are hallmarks of the moral foundations of the members of this paramilitary group, and that these values, in comparison with a tendency toward agreeableness and away from high neuroticism is significantly different from the general population and can serve as a cognitive profile for paramilitary motivations.

Lastly, when testing H3, we found evidence that there is a link between two key beliefs central to the experience and identity of Slovenskí Branci that are affecting levels of identity fusion. These are (1) the extent to which they are offended by the comparisons between nationalism and fascism/racism and (2) the belief that Slovakia should be self-sufficient. In both cases, these variables have positive effects on levels of identity fusion. Of the seven social variables that were included in our model (all on beliefs about different aspects of Slovak life/culture), those were the only two significant predictors.

Some limitations of this research must also be considered. First, further validation of our results would require replication. Central and Eastern Europe have a specific historical experience, related to World War II and the subsequent communist era, that led to varying national strategies when dealing with non-state paramilitaries. Such strategies have ranged from mostly positive attempts to support civilian participation on defense (e.g., Poland and the Baltic states) to indifferent or slightly negative stances (e.g., Slovakia and the Czech Republic). However, local paramilitaries tend to react to current cultural and political developments, and the further removed citizens are from formative historical experiences, the more relevant the current world events become for them. As formative events fade, and current events take focus, the historical experience becomes just one of the factors that influence the psychological and cognitive profile of individuals and groups. It would be interesting to see if profiles we found in our sample (unlike profiles of extremist or terrorist groups, which tend to be low on agreeableness) can be found elsewhere (e.g., in American militia groups, Polish pro-defense organizations, or Russian military-patriotic clubs).

Second, future research may want to investigate whether the outliers we found with the purity indicator of MFT and neuroticism in BFPT are the result of long-term membership inside the group, where the environment “acts upon” the member whose neuroticism decreases [30, 31], or whether the group tends to attract—or at least retain as long-term members—individuals naturally low on neuroticism (i.e., whether those with higher neuroticism do not join, or tend to leave quickly).

A third limitation, common to such studies, has to do with measurement reliability. The Big 5 Personality traits and the Moral Foundations questionnaires are short questionnaires that are practical in the field. However, measuring complex personality and cognitive traits in this way, rather than using lengthier questionnaires (of the sort often administered in lab settings rather than paramilitary field training exercises), might limit the precision and nuance of the outcomes. This calls for interdisciplinary work between psychologists and field anthropologists to develop field-ready and reliable measures that can be rigorously psychometrically validated for use in contexts such as the one in the study reported here.

Finally, the methods used to access the unique data described here might also be considered a limitation of the study. Field research itself can change the behavior in members of a group, who may become self-aware while they are being observed. The authors believe this concern can be addressed in two ways. First, the field research took place over several years, after members were acclimated to the field researcher, and saw that their objective is not to write a sensational newspaper article, but scholarly analysis. Gaining this trust required leveraging the method of participant observation, i.e., fully embedding into the environment of the paramilitary organization (apart from training itself). This includes one of the authors participating in the weeklong trainings, which included sleeping in the same tents, eating the same (outdoor-cooked) food, enduring the same weather, wearing the same clothes (except, for ethical reasons, wearing a badge that identified the researcher as a non-member), and becoming sick with the same stomach problems as any other member when an illness swept through the camp. This built a level of trust between the researcher and the members of the group that lessened the effects of biases (e.g., members answering in ways they felt the field researcher desired). In addition, the mental and physical demands during the research periods made it difficult for participants to uphold any possible sustained pretenses.

Furthermore, and perhaps most importantly, members are generally proud of their beliefs and values that guide their lives–which is why they joined a paramilitary group in the first place–so they are not ashamed of their beliefs and do not tend to lie about them. Self-censorship tends to come in the form of toning down their expressions and sentiments for convenience or politeness. It is also hard to uphold a pretense across 100+ members that the authors talked to over several years, especially when interviewing former members as well. For these reasons, we believe that these data are relatively reliable despite the potential limitations outlined here.

## Conclusion

What have we learned about the nature of paramilitary organizations by examining Slovenskí Branci? First, there is a suite of complex factors that are known to predict socio-political values, such as the moral foundations measured in this study, for which paramilitaries do not fit as neatly as one might expect; this is specifically the case concerning the unexpectedly low importance of purity reported in our data. This may be the result of the dynamic and complex socio-political environments that any particular paramilitary aims to affect. For example, regarding the socio-historical context of our current sample: Slovenskí Branci have evolved from their initial far-right leanings to a more complex nationalist organization and its membership’s psychological makeup as measured in this study supports this observation. With our current data we cannot explain the reasons underpinning this change. However, studying moral foundations and personality traits over time could add new insight to our knowledge of armed non-state actors and their motivations. The knowledge provided by the current study is made all the more valuable since similar data are extremely scarce due to limited access to such groups.

Second, this study contributes to our understanding of what types of people might be attracted to join paramilitary groups and what motivates them to remain once they become members. We found that, at least in the case of Slovenski Branci, that demographic factors are not good predictors of paramilitary action. Members are drawn from across the demographic spectrum in terms of age, ethnicity/race, rural/urban, income bracket, and marital status. The only useful demographic would be gender, as the group is primarily male, however, this is not theoretically interesting as males are more likely to fight for their group across all primate groups [32]. Our results suggest that paramilitary groups can be attractive to a wider segment of population than previously expected, since their psychological profile does not align with the typical extremist profile. To further support this, future research needs to be done on samples of the general population, former members of extremist and terrorist groups, and with members of active-duty military.

Paramilitary groups stylized as self-defense forces often occupy a “grey zone” between organizations that are directly fighting against the state and organizations that clearly serve to protect the state. Such groups do clearly challenge states’ monopoly on the legitimate use of violence. This situates paramilitary organizations as a potential—but not obviously acute—threat to peace. They are typically viewed as benign enough to be tolerated by a state’s security apparatus even though–or perhaps because–of the way in which they provide a social outlet for citizens who either do not trust state institutions or who desire extra support networks in addition to the state. All of this complicates the task of politicians and policy makers who are responsible for maintaining peace within (and between) national borders. Our research provides new data, based on a new and detailed psychometric survey made possible by unique longitudinal field research access, that can aid a wide range of stakeholders as they struggle to understand the motivations of members of non-state armed organizations and their potential threat to peace.
